# Low-Toxicity and High-Stability Fluorescence Sensor for the Selective, Rapid, and Visual Detection Tetracycline in Food Samples

**DOI:** 10.3390/molecules29245888

**Published:** 2024-12-13

**Authors:** Jixiang Wang, Yaowei Qin, Yue Ma, Minjia Meng, Yeqing Xu

**Affiliations:** 1 School of Chemistry and Chemical Engineering, Jiangsu University, Zhenjiang 212013, China; 2 State Key Laboratory of Molecular Engineering of Polymers, Laboratory of Advanced Materials, Shanghai Key Lab of Molecular Catalysis and Innovative Materials, Department of Chemistry, Fudan University, Shanghai 200438, China

**Keywords:** low toxicity, tetracycline, ratiometric fluorescent molecularly imprinted sensor, visual detection

## Abstract

With the development and improvement of analysis and detection systems, low-toxicity and harmless detection systems have received much attention, especially in the field of food detection. In this paper, a low-toxicity dual-emission molecularly imprinted fluorescence sensor (CdTe QDs@SiO_2_/N-CDs@MIPs) was successfully designed for highly selective recognition and visual detection of tetracycline (TC) in food samples. Specifically, the non-toxic blue-emission N-doped carbon dots (N-CDs) with high luminous performance acted as the response signals to contact TC via the covalent bond between amino and carboxyl groups. The red-emission CdTe quantum dots (CdTe QDs) were coated in silica nanospheres as stable reference signals, which effectively avoided the direct contact of CdTe QDs. Under optimum conditions, CdTe QDs@SiO_2_/N-CDs@MIPs had a rapid response within 1.0 min to TC, and the detection limit of CdTe QDs@SiO_2_/N-CDs@MIPs was calculated at 0.846 μM in the linear range of 0–140 μM. In complex environments, the CdTe QDs@SiO_2_/N-CDs@MIPs also exhibited excellent capabilities for the selective, rapid, and visual detection of TC. Furthermore, the accuracy of CdTe QDs@SiO_2_/N-CDs@MIPs to detect TC was verified by the HPLC method, and satisfactory results were obtained. Moreover, CdTe QDs@SiO_2_/N-CDs@MIPs showed a satisfactory recovery when measuring TC in milk and egg samples. This work provided an ideal approach for low-toxicity fluorescence sensor design and application.

## 1. Introduction

Tetracycline (TC) is a broad-spectrum antibiotic, which shows an inhibitory effect on most Gram-positive and Gram-negative bacteria [1,2]. High concentrations of TC have bactericidal effects and better effects on Gram-negative bacilli; it can inhibit rickettsia, trachoma virus, etc. [3,4,5]. Therefore, TC has been widely used in the treatment of bacterial infections in humans and animals for a long time [6,7,8]. However, such widespread use of TC will lead to the increased level of drug-resistant antibiotic residues in the human living environment, then appear in food, and transfer to humans through the food chain. Finally, TC residues will result in adverse reactions, such as effects on the digestive tract, liver damage, kidney damage, impacts on the development of teeth and bones, local irritation, allergic reactions, intestinal flora imbalance, and the formation of complexes that hinder utilization [9,10]. Therefore, it is urgent to develop a fast, simple, and sensitive method to directly detect TC in food samples.

The molecular imprinting fluorescence analysis method, equipped with the sensitivity of fluorescence analysis and the selectivity of molecular imprinting technology (MIT), is a great choice to realize the effective detection of TC. Yang et al. designed a novel fluorescence sensor that combined carbon dots (CDs) with molecularly imprinted polymers (MIPs); the MIPs were successfully used to detect TC in fish samples with high sensitivity and accuracy [11]. On the other hand, there were many studies committed to improving the real-time detection of targets, such as the one conducted by Chen et al., in which a ratiometric fluorescent probe was constructed based on CdTe QDs and silicon nanoparticles (SiNPs); the fluorescence of CdTe QDs was quenched by Hg^2+^ through ion-binding and electron transfer processes and recovered after the addition of glutathione. This fluorescence sensor was applied to detect azodicarbonamide (ADA) in flour rapidly [12]. Thus, the introduction of ratiometric fluorescence makes the visual detection of targets possible.

Generally, single-emission sensors often experience interference from various factors, including changes in concentration, non-uniform sensor distribution, the efficiency of the instruments, and the environmental conditions present in intricate samples [13,14,15,16]. In contrast, a ratio fluorescence sensor involves measuring two or more well-resolved wavelengths of fluorescent signals simultaneously and then calculating their intensity ratios to circumvent these adverse effects, so as to determine the approximate range of the target substance content [17,18,19,20]. As expected, the combination of ratiometric fluorescence sensors and MIT greatly expands the application range of ratio fluorescence sensors. In recent years, molecularly imprinted ratiometric fluorescence sensors have been widely used in the detection of targets (such as antibiotics, phenolic compounds, drug residues, protein targets, and ionic targets, etc.) [21,22,23,24,25]. In the latest work of our group [26], a ratiometric fluorescence sensor was successfully synthesized to detect dopamine, in which CdTe QDs and CDs were used as the response signal and reference signal, respectively. Inevitably, CdTe QDs are harmful to the human body, but the fluorescence performance of CdTe QDs is superior to other water-soluble quantum dots. In view of its excellent fluorescence performance, in this experiment, CdTe QDs were used as a stable core to construct a ratiometric fluorescence sensor with harmless CDs.

CDs, which are an important member of the quantum dot family, have been rapidly developed for their outstanding properties, such as non-toxicity, water solubility, biocompatibility, simple synthetic routes, high photochemical stability, and tunable excitation and emission spectra [27,28,29]. CDs are expected to become the next generation of green nanomaterials that can replace traditional fluorophores, such as organic dyes and conjugated polymers [30,31,32]. However, the relatively low fluorescence quantum yield of CDs still limits their application in the field of fluorescence sensing. Studies have demonstrated heteroatom doping as a relatively efficient and simpler method to improve fluorescence properties [33,34].

Based on the above analysis, non-toxic N-CDs were chosen to be the response signal to realize the detection of TC in food samples. CdTe QDs were coated in silica to avoid leaks and act as the reference signal. Thus, a low-toxicity molecularly imprinted ratiometric fluorescence sensor (CdTe QDs@SiO_2_/N-CDs@MIPs) was successfully synthesized via precipitation polymerization for TC residue detection. Under optimal detection conditions, the fluorescence intensity of N-CDs decreased quickly with the increasing concentration of TC, due to the matched imprinting sites in CdTe QDs@SiO_2_/N-CDs@MIPs, while the fluorescence intensity of CdTe QDs remained relatively stable. Furthermore, the HPLC analysis forcefully proved that CdTe QDs@SiO_2_/N-CDs@MIPs could be effectively applied in the rapid and accurate detection of TC in actual samples. In addition, CdTe QDs@SiO_2_/N-CDs@MIPs presented different colors from blue to magenta under different TC concentrations, which were easy to distinguish. This work provided an ideal approach for designing stable and low-toxicity ratiometric fluorescence sensors for the detection of hazardous substances.

## 2. Results

### 2.1. Characterization of CdTe QDs@SiO_2_/N-CDs@MIPs

**Figure 1:**
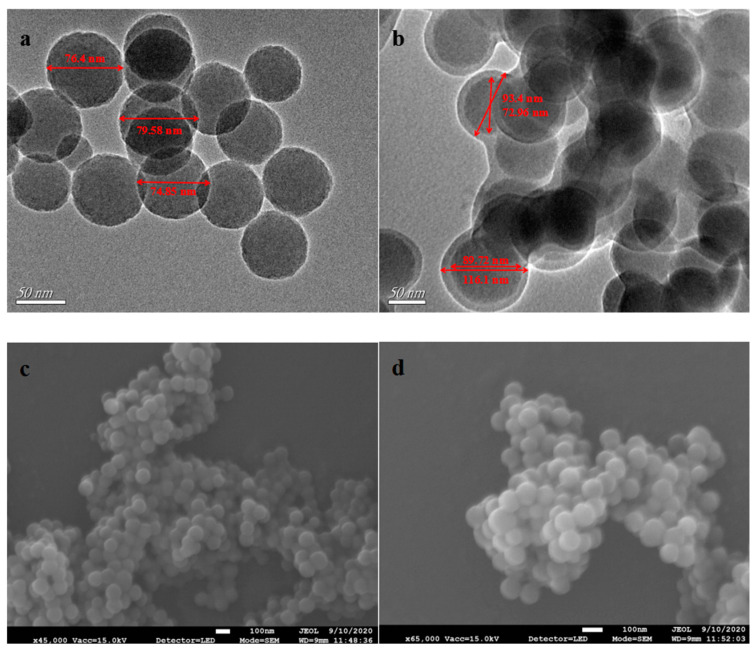
TEM images of CdTe QDs@SiO_2_ (**a**) and CdTe QDs@SiO_2_/N-CDs@MIPs (**b**); SEM images of CdTe QDs@SiO_2_/N-CDs@MIPs (**c**,**d**).

The synthesized CdTe QDs@SiO_2_/N-CDs@MIPs were characterized by thermal field emission scanning electron microscope (SEM), transmission electron microscope (TEM), and Fourier transform infrared spectrometer (FT-IR). Figure 1a is the TEM image of CdTe QDs@SiO_2_ with strong monodispersity. The particle size of CdTe QDs@SiO_2_ measured by the software ImageJ (https://imagej.net/software/fiji/downloads, accessed on 11 December 2024) was 80 ± 10 nm. Figure 1b is the TEM image of CdTe QDs@SiO_2_/N-CDs@MIPs. It can be clearly seen from the figure that a polymer layer was formed with a thickness of about 11 nm on the surface of CdTe QDs@SiO_2_, suggesting an imprinting layer had taken shape. According to the previous research of our group, the thinner imprinting layer could significantly reduce the detection time of the target analyte [35,36]. Figure 1c,d are the SEM images of CdTe QDs@SiO_2_/N-CDs@MIPs. It can be seen from the figure that the CdTe QDs@SiO_2_/N-CDs@MIPs presented regular and uniform spheres.

**Figure 2:**
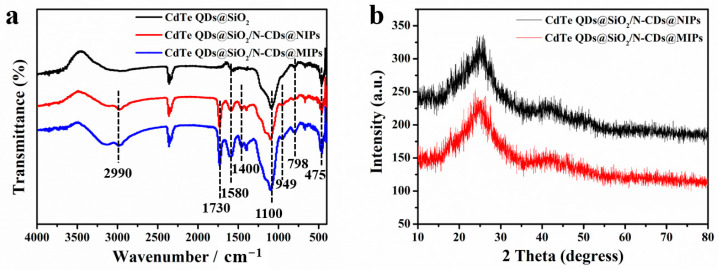
FT-IR spectra (**a**) of CdTe QDs@SiO_2_ (black line), CdTe QDs@SiO_2_/N-CDs@NIPs (red line), and CdTe QDs@SiO_2_/N-CDs@MIPs (blue line); XRD patterns (**b**) of CdTe QDs@SiO_2_/N-CDs@NIPs (black line) and CdTe QDs@SiO_2_/N-CDs@MIPs (red line).

In order to further characterize the synthesized CdTe QDs@SiO_2_/N-CDs@MIPs, FT-IR was performed. Figure 2a shows the FT-IR spectra of CdTe QDs@SiO_2_ (black line), CdTe QDs@SiO_2_/N-CDs@NIPs (red line), and CdTe QDs@SiO_2_/N-CDs@MIPs (blue line). As displayed in Figure 2a, the broad absorption peak of 1110 cm^−1^ indicates the asymmetric stretching of Si-O-Si, the absorption peaks at 475 cm^−1^ and 798 cm^−1^ indicate the flexural vibration peaks of Si-O, and the absorption peak of Si-H appears at 949 cm^−1^, which jointly prove the existence of SiO_2_. The C=O stretching vibration at 1730 cm^−1^ proves the existence of EGDMA. The characteristic peaks at 1400 cm^−1^ and 1580 cm^−1^ are the flexural vibration peaks of N-H and C-H, respectively, which indicate the presence of functional monomer AM in the polymers. The characteristic peak at 2990 cm^−1^ indicates C-H tensile vibration. The experimental characterization results imply that CdTe QDs@SiO_2_/N-CDs@MIPs were successfully prepared. Figure 2b shows the XRD diffraction peak pattern of CdTe QDs@SiO_2_/N-CDs@MIPs and CdTe QDs@SiO_2_/N-CDs@NIPs. The results demonstrate that the crystal structure of CdTe QDs@SiO2/N-CDs@MIPs did not change after imprinting.

**Figure 3:**
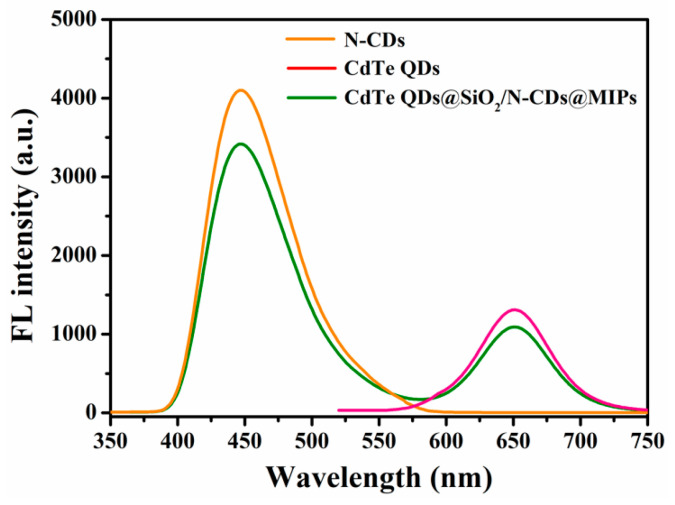
Fluorescence spectra of N-CDs (orange line), CdTe QDs (pink line), and CdTe QDs@SiO_2_/N-CDs@MIPs (green line).

The corresponding fluorescence spectra of N-CDs, CdTe QDs, and CdTe QDs@SiO_2_/N-CDs@MIPs are shown in Figure 3. The CdTe QDs@SiO_2_/N-CDs@MIPs show obvious emission peaks at both 447 nm and 651 nm, respectively, overlapping with the emission peaks of single-emission N-CDs and CdTe QDs. Therefore, the synthesized CdTe QDs@SiO_2_/N-CDs@MIPs display both the emission peaks of N-CDs and CdTe QDs. The fluorescence spectra indicate that the synthesized CdTe QDs@SiO_2_/N-CDs@MIPs had the characteristics of dual emission.

### 2.2. Optimization of Detection Conditions

Before using CdTe QDs@SiO_2_/N-CDs@MIPs to detect TC, the detection conditions were optimized, including stability, reaction time, and pH value.

**Figure 4:**
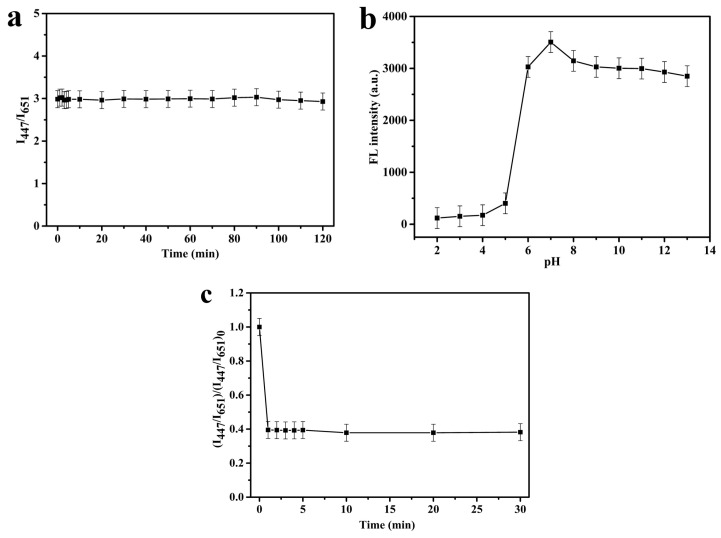
Fluorescence intensity ratios of the CdTe QDs@SiO_2_/N-CDs@MIPs solution over 120 min (**a**); pH effect on the fluorescence intensity of CdTe QDs@SiO_2_/N-CDs@MIPs (**b**); the effect of 70 μM TC on the fluorescence intensity of CdTe QDs@SiO_2_/N-CDs@MIPs over time (**c**).

Firstly, the stability of CdTe QDs@SiO_2_/N-CDs@MIPs was studied. The fluorescence stability of CdTe QDs@SiO_2_/N-CDs@MIPs solution was determined through the detection of fluorescence intensities. At room temperature, the fluorescence intensities of CdTe QDs@SiO_2_/N-CDs@MIPs at different moments were studied and recorded. As shown in Figure 4a, the fluorescence intensity ratio (I_447_/I_651_) of the CdTe QDs@SiO_2_/N-CDs@MIPs was measured and calculated repeatedly within 120 min. The experimental results indicate that the CdTe QDs@SiO_2_/N-CDs@MIPs prepared in this experiment had good optical stability, and the changes in fluorescence intensity in the subsequent detection were attributed to the addition of the target.

Secondly, the influence of the pH value in the range from 1.0 to 14 on the fluorescence intensity of CdTe QDs@SiO_2_/N-CDs@MIPs was studied. Generally, the pH value of the solution had a greater impact on the fluorescence performance of CdTe QDs@SiO_2_/N-CDs@MIPs. The changes in the fluorescence intensity of the CdTe QDs@SiO_2_/N-CDs@MIPs solution at 447 nm with different pH values are shown in Figure 4b. It can be seen in the figure that when the pH value was 7.0, the fluorescence intensity of the solution reached its maximum value. When the solution was too acidic, a significant decline was observed in fluorescence intensity; when the solution was too alkaline, the fluorescence intensity of the solution decreased to a certain value and remained relatively stable. It was shown that the neutral environment was more suitable for CdTe QDs@SiO_2_/N-CDs@MIPs to detect TC. Because all tests were carried out in water, a small amount of TC would not have had a major impact on the pH of the water, so this experiment could be used to test for TC without adjusting the pH value.

Finally, in order to determine the best response time of CdTe QDs@SiO_2_/N-CDs@MIPs to TC, 70 μM of TC was added to the CdTe QDs@SiO_2_/N-CDs@MIPs solution. Then, the fluorescence intensities were recorded and the ratios of (I_447_/I_651_)/(I_447_/I_651_)_0_ at different times were calculated. The experimental results are displayed in Figure 4c. The fluorescence intensity of the solution initially decreased rapidly within 1.0 min. When the detection time reached 1.0 min, the fluorescence intensities of CdTe QDs@SiO_2_/N-CDs@MIPs remained almost unchanged with the continuation of time. Thus, 1.0 min was selected as the best response time of CdTe QDs@SiO_2_/N-CDs@MIPs to TC, which was superior to most molecular imprinting sensors [37,38,39], and it clearly demonstrated that a thinner imprinting layer could speed up the response time of the target.

### 2.3. Fluorescence Determination Capability

Under ideal conditions, the study investigated the fluorescence detection capabilities of CdTe QDs@SiO_2_/N-CDs@MIPs for various concentrations of tetracycline (TC) and its ability to be visually detected. At ambient temperature, solutions of TC at different concentrations were combined with CdTe QDs@SiO_2_/N-CDs@MIPs to achieve a predetermined volume, creating a test sample solution that was subsequently poured into a quartz cuvette for fluorescence analysis.

**Figure 5:**
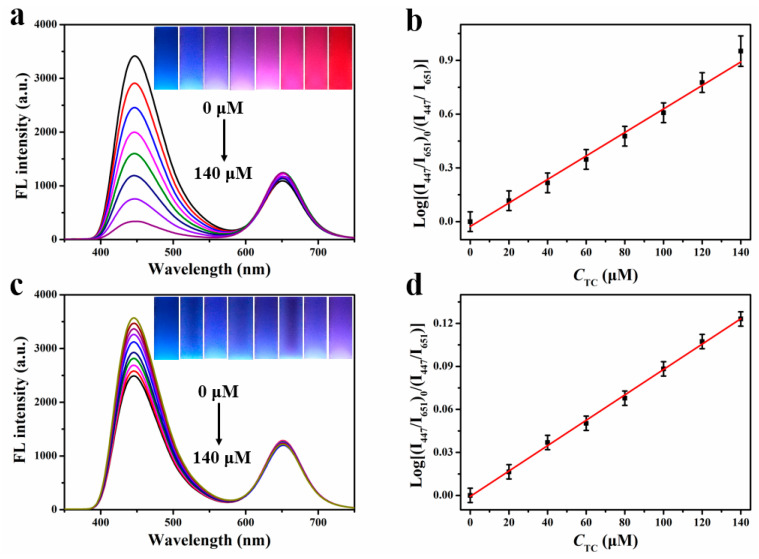
Fluorescence spectra and visual diagrams (inset) of CdTe QDs@SiO_2_/N-CDs@MIPs solution (**a**) and CdTe QDs@SiO_2_/N-CDs@NIPs solution (**c**) with different concentrations of TC; linear relationship diagram of different concentrations of TC added to CdTe QDs@SiO_2_/N-CDs@MIPs solution (**b**) and CdTe QDs@SiO_2_/N-CDs@NIPs solution (**d**).

After the addition of TC, the fluorescence intensity of blue-emission N-CDs at 447 nm gradually decreased with the increase in TC concentration, while the fluorescence intensity of red-emission CdTe QDs at 651 nm remained relatively stable. As displayed in Figure 5a, with the TC concentration increased from 0 μM to 140 μM, the fluorescence intensity of CdTe QDs@SiO_2_/N-CDs@MIPs at 447 nm continuously decreased. By calculating the fluorescence intensity ratio between 447 nm and 651 nm, the linear relationship between the fluorescence intensity ratios of CdTe QDs@SiO_2_/N-CDs@MIPs and TC concentrations was obtained, as described in Figure 5b. The linear relationship was calculated as follows: Log[(I_447_/I_651_)_0_/(I_447_/I_651_)] = 0.00656C_TC_ − 0.0268 (R^2^ = 0.99164), where “C_TC_“ is the concentration of TC, and “I” is the fluorescence intensity of CdTe QDs@SiO_2_/N-CDs@MIPs. Therefore, CdTe QDs@SiO_2_/N-CDs@MIPs could quantitatively detect TC at a concentration range of 0–140 μM, and the detection limit (3σ/k) was calculated at 0.846 μM, where “k” is the slope of the linear fitting equation, and “σ” is the standard deviation of the blank measurement after 10 detections (*n* = 10). The inset of Figure 5a shows the pictures of CdTe QDs@SiO_2_/N-CDs@MIPs with increasing concentrations of TC (from left to right); the color of CdTe QDs@SiO_2_/N-CDs@MIPs changed from blue to red as the TC concentration increased, which was clearly discernible to the naked eye.

In order to more intuitively prove the specific recognition ability of CdTe QDs@SiO_2_/N-CDs@MIPs to TC, CdTe QDs@SiO_2_/N-CDs@NIPs were used as the comparison in the experiment. Figure 5c shows the detection results of CdTe QDs@SiO_2_/N-CDs@NIPs for different concentrations of TC. It can be seen that with the increasing concentration of TC in the test solution, the fluorescence intensity ratio of CdTe QDs@SiO_2_/N-CDs@NIPs decreased slightly, the quenching degree was not large, and the color changes (inset in Figure 5c) of CdTe QDs@SiO_2_/N-CDs@NIPs were not obvious enough to be distinguishable. The main reason for this result was that there were no binding sites matching with TC in CdTe QDs@SiO_2_/N-CDs@NIPs, and the template molecule TC could not interact with N-CDs through the polymerization layer and quench them. Figure 5d shows the linear fitting equation of CdTe QDs@SiO_2_/N-CDs@NIPs; the linear relationship was calculated as follows: Log[(I_447_/I_651_)_0_/(I_447_/I_651_)] = 1.358 × 10^−5^C_TC_ − 7.021 × 10^−4^ (R^2^ = 0.99836). Compared with CdTe QDs@SiO_2_/N-CDs@NIPs, CdTe QDs@SiO_2_/N-CDs@MIPs exhibited not only a significant degree of quenching but also an outstanding effect in visual detection.

### 2.4. Specific Recognition of TC by CdTe QDs@SiO_2_/N-CDs@MIPs

**Figure 6:**
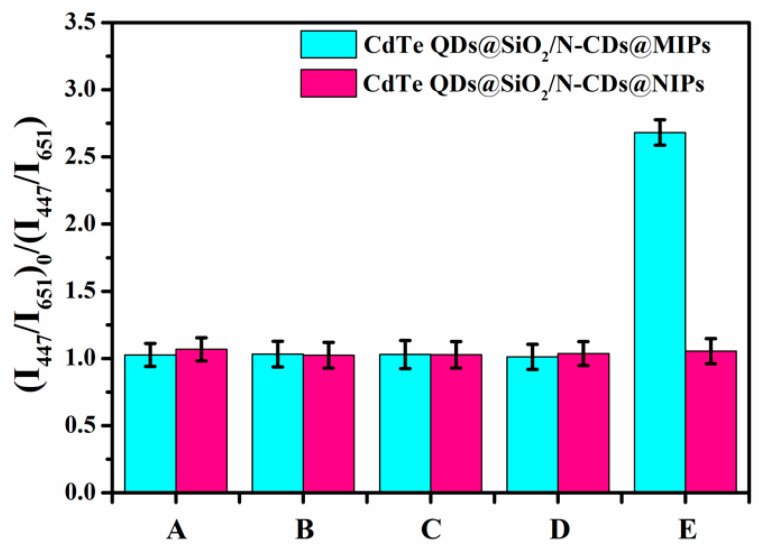
The relative change rate of the ratio of CdTe QDs@SiO_2_/N-CDs@MIPs and CdTe QDs@SiO_2_/N-CDs@NIPs to the fluorescence intensity of TC and similar drugs (amoxicillin (A), erythromycin (B), streptomycin (C), azithromycin (D), and tetracycline (E)) under the same concentration conditions.

In order to study the selectivity of CdTe QDs@SiO_2_/N-CDs@MIPs to TC, the effects of tetracycline (E) and other similar drugs (amoxicillin (A), erythromycin (B), streptomycin (C), and azithromycin (D)) on CdTe QDs@SiO_2_/N-CDs@MIPs were explored. The fluorescence intensities of CdTe QDs@SiO_2_/N-CDs@MIPs with the same concentrations of these drugs were recorded and the intensity ratios (I_447_/I_651_) were calculated to analyze the selection of CdTe QDs@SiO_2_/N-CDs@MIPs. It can be seen from Figure 6 that when TC was present, the value of (I_447_/I_651_)_0_/(I_447_/I_651_) was 2.77. Under the same drug concentration, TC had the greatest quenching effect on the fluorescence intensity of CdTe QDs@SiO_2_/N-CDs@MIPs compared with other similar drugs. This phenomenon could be explained by the existence of a large number of binding sites matching with TC in the imprinted polymer layer. Upon the addition of TC, the specific recognition sites on the CdTe QDs@SiO_2_/N-CDs@MIPs rapidly rebound with TC, resulting in a swift electron transfer and the consequent fluorescence quenching of the N-CDs. In contrast, drugs similar to TC are unable to establish a complementary interaction with the binding sites of the CdTe QDs@SiO_2_/N-CDs@MIPs due to discrepancies in their spatial configurations and molecular dimensions compared to the template molecule TC. Therefore, they could not be adsorbed completely into the polymer layer by CdTe QDs@SiO_2_/N-CDs@MIPs.

Concurrently, the selectivity of CdTe QDs@SiO_2_/N-CDs@NIPs was examined under identical conditions. As depicted in Figure 6, it was observed that TC and analogous drugs exerted negligible influence on the fluorescence intensity of CdTe QDs@SiO_2_/N-CDs@NIPs. These findings further confirmed that CdTe QDs@SiO_2_/N-CDs@MIPs possess a specific recognition ability for TC, distinguishing them from other similar drugs that do not interact with the binding sites in a specific manner. It also proved that there were no binding sites that could specifically recognize TC in CdTe QDs@SiO_2_/N-CDs@NIPs.

### 2.5. Ions Inference Study

**Figure 7:**
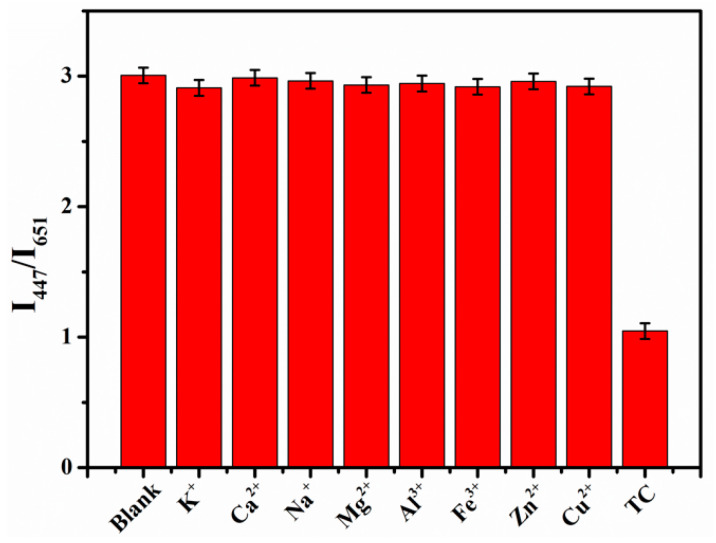
The fluorescence intensity ratio (I_447_/I_651_) of CdTe QDs@SiO_2_/N-CDs@MIPs after addition of 100 μM of K^+^, Mg^2+^, Al^3+^, Fe^3+^, Zn^2+^, Cu^2+^, and TC, and 50 mM of Ca^2+^ and Na^+^.

The first prerequisite for applying CdTe QDs@SiO_2_/N-CDs@MIPs to actual detection was to eliminate the interference of common metal ions in the actual environment. Therefore, the influence of some common metal ions on CdTe QDs@SiO_2_/N-CDs@MIPs was studied. Specifically, 100 μM of K^+^, Mg^2+^, Al^3+^, Fe^3+^, Zn^2+^, Cu^2+^, and TC, and 50 mM of Ca^2+^ and Na^+^ (there is a relatively high content of calcium and sodium in milk) were added to the test solution of CdTe QDs@SiO_2_/N-CDs@MIPs; the fluorescence intensity ratios of the blank solution and solutions with metal ions were recorded and calculated, as shown in Figure 7. It can be seen that the metal ions had almost no effect on fluorescence intensity changes, which indicates that CdTe QDs@SiO_2_/N-CDs@MIPs could be applied for detecting TC in actual samples.

### 2.6. Fluorescence Quenching Mechanism

**Figure 8:**
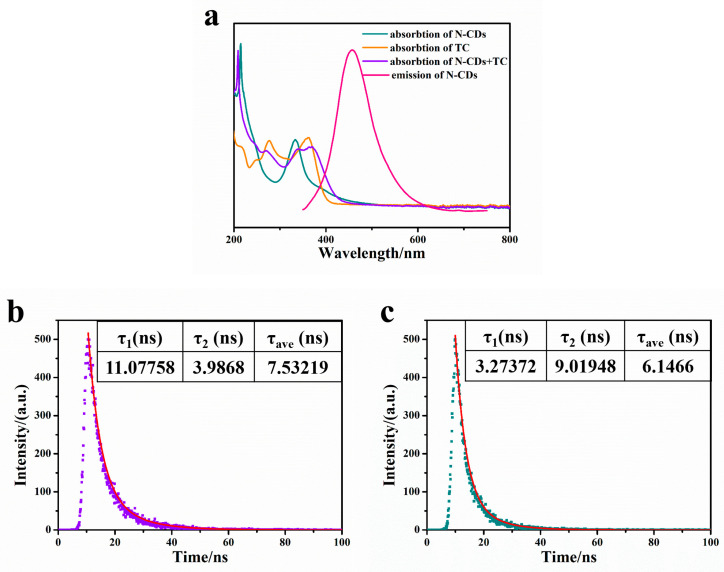
(**a**) The absorption spectra of N-CDs (green line), TC (orange line), the mixture of N-CDs and TC (purple line), and the fluorescence emission spectrum of N-CDs (pink line); the transient fluorescence lifetime diagrams of N-CDs (**b**) and the mixture of N-CDs and TC (**c**).

To further explore the interaction between N-CDs and TC, as well as its quenching mechanism, the corresponding UV absorption and transient fluorescence lifetime were detected and are displayed in Figure 8. As exhibited in Figure 8a, there was no obvious shift in the UV absorption peak positions of N-CDs (green line) and TC (orange line) before and after mixing, in which the UV absorption of the mixture is shown as the purple line in Figure 8a. Thus, the possibility of forming a complex between them was ruled out, and the quenching mechanism may not be the internal filtration effect [40]. The fluorescence emission of N-CDs (pink line) and the UV absorption of TC also excluded the possibility of fluorescence resonance energy transfer [41]. Then, it can be seen from Figure 8b,c that the original transient fluorescence lifetime of N-CDs without TC was 7.53 ns, the transient fluorescence lifetime of N-CDs with TC was 6.15 ns, and the inequality τ_0_/τ_1_ ≠ 1 suggested that static quenching was not the operative mechanism. Consequently, the most likely quenching mechanism for the interaction between N-CDs and TC was dynamic quenching [42], with the measured linear equation aligning with the dynamic Stern–Volmer quenching model [43].

### 2.7. Actual Sample Detection and Recovery Experiment

**Figure 9:**
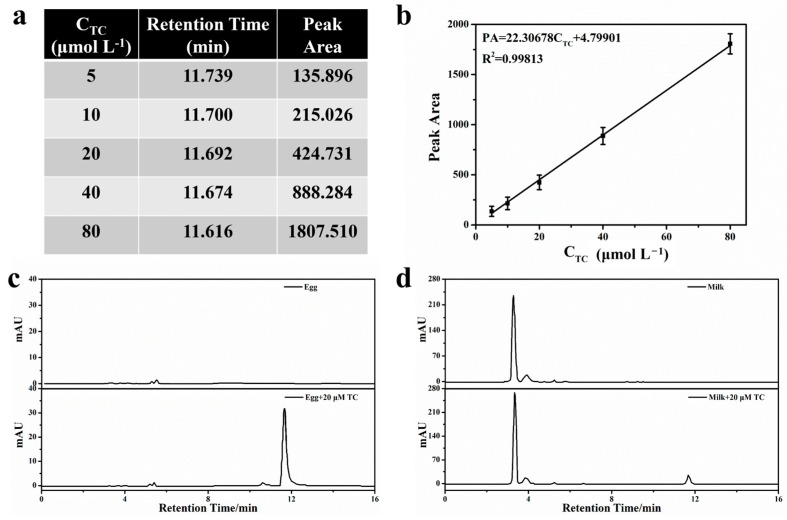
(**a**,**b**) The parameters of HPLC-UV results and the corresponding calibration curve for TC; (**c**) the HPLC-UV profiles of eggs and eggs spiked with 20 μM TC; (**d**) the HPLC-UV profiles of milk and milk spiked with 20 μM TC.

In this experiment, CdTe QDs@SiO_2_/N-CDs@MIPs were used to detect the content of TC in the actual milk and egg samples by using the standard addition recovery method. The HPLC analysis method was applied to verify the practicability of the established analytical method. The TC standard solution was added to the reagent sample, and then the standard addition recovery method was used to carry out the addition recovery experiment; the experimental results of the fluorescence analysis method and the HPLC method are shown in Table 1. It can be seen that the detection results of TC in milk and eggs by CdTe QDs@SiO_2_/N-CDs@MIPs were much in line with those obtained by the HPLC method. The specific parameters and spectra of TC detection by the HPLC method are shown in Figure 9. Meanwhile, compared with other detection methods, which are summarized in Table 2, ratio fluorescence analysis of this work exhibited a satisfactory detection speed and detection limit. To summarize, the obtained results strongly demonstrate that the CdTe QDs@SiO_2_/N-CDs@MIPs proposed in this work can be successfully applied to the fast and accurate analysis of actual samples.

## 3. Materials and Methods

### 3.1. Materials

NaBH_4_, Tellurium, CdCl_2_·2.5H_2_O, NaOH, hexanol, TritonX-100, cyclohexane, acetone, ethanol, acetonitrile, anhydrous citric acid, urea, tetraethyl orthosilicate (TEOS), ammonium hydroxide, KH570, Toluene, Acrylamide (AM), Thioglycolic acid (TGA), Ethylene dimethacrylate (EGDMA), and azodiisobutyronitrile (AIBN) were all purchased from Aladdin Reagent Company (Shanghai, China). Acetonitrile (HPLC) and acetic acid (HPLC) were bought from Guoyao Chemical Reagent (Shanghai, China). Deionized water was used throughout the experiment.

### 3.2. Synthesis

#### 3.2.1. Synthesis of CdTe QDs and N-CDs

CdTe QDs and N-CDs were prepared according to our previous reports [25].

CdTe QDs were prepared by the hydrothermal method. Firstly, the precursor was prepared by the following: 51 mg of tellurium powder, 100 mg of NaBH_4_, and 2.0 mL of H_2_O were added in a centrifuge tube with needle, and the mixture was ultrasonically dispersed until the color turned transparent.

Next, CdTe QDs were synthesized through the reflow method, and 365.38 mg of CdCl_2_·2.5H_2_O, 176 μL of TGA, and 98 mL of H_2_O were added into a 250 mL three-necked flask. The pH was adjusted to 11.2 in a 1.0 mol·L^−1^ NaOH solution, and the solution was bubbled with N_2_ for 30 min. Finally, the precursor solution was quickly added. The reflux reaction was kept for 7.0 d under nitrogen atmosphere at 130 °C, and red-emission CdTe QDs were collected for later use.

The detail steps of CDs preparation were as follows: 0.5 g of anhydrous citric acid and 0.5 g of urea were dissolved in 10 mL of H_2_O, and subsequently, the mixture was poured into a stainless steel vessel lined with Teflon, and then subjected to hydrothermal processing at 160 °C for 4.0 h in an oven. Once cooled to ambient temperature, the resulting dark green solution was precipitated with acetone three times, subsequently dried using a freeze dryer, and then redissolved in 10 mL of water.

#### 3.2.2. Preparation of CdTe QDs@SiO_2_

A total of 1.0 mL of CdTe QDs, 7.5 mL of cyclohexane, 1.8 mL of 1-hexanol, and 1.77 mL of TritonX-100 were added in a 25 mL round-bottom flask in sequence and mixed uniformly. Then, 400 μL of H_2_O and 240 μL of ammonium hydroxide were added and stirred magnetically for 30 min. Finally, the reaction was magnetically stirred at room temperature in the dark for 24 h after the addition of 100 μL of TEOS. After the reaction, the emulsion was demulsified with an equal volume of acetone, centrifuged and washed with ethanol, dried, and kept for later use.

#### 3.2.3. Synthesis of CdTe QDs@SiO_2_/N-CDs@MIPs

Functional modification of CdTe QDs@SiO_2_ was performed: 100 mg of CdTe QDs@SiO_2_ and 1.0 mL of KH570 were dissolved in 50 mL of toluene and heated at 90 °C for 24 h. The final product was centrifuged, washed with ethanol, and dried for further use.

**Scheme 1:**
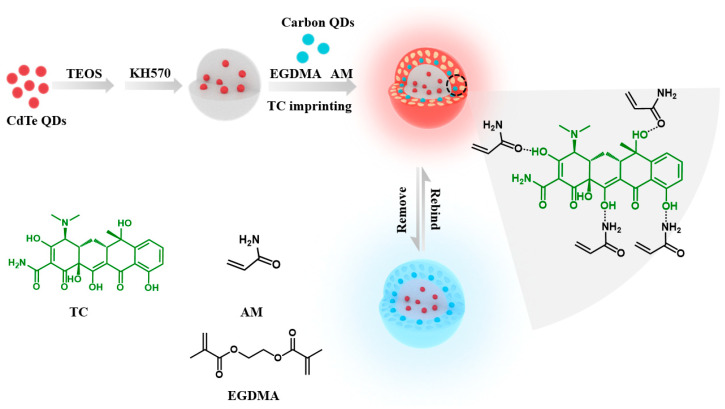
The preparation flow chart of CdTe QDs@SiO_2_/N-CDs@MIPs.

The preparation flow chart of CdTe QDs@SiO_2_/N-CDs@MIPs is shown in Scheme 1 according to the precipitation polymerization method. Specifically, 100 mg of CdTe QDs@SiO_2_-KH570 was first added and used as both the reference signal and the carrier of N-CDs. Then, 1.0 mL of N-CDs, 9.47 mg of AM, 44 mg of TC, and 132 μL of EGDMA were dissolved in 60 mL of ethanol. Nitrogen was introduced into the reaction for 30 min to maintain an oxygen-free environment, after which the initiator AIBN was added, and nitrogen was introduced continuously for another 20 min. After that, the reaction was kept at 50 °C for 6.0 h and up to 60 °C for another 24 h. During the washing process, CdTe QDs@SiO_2_/N-CDs@MIPs were eluted with 100 mL acetic acid/methanol (10/90, *v*/*v*) by a soxhlet extractor for 2 days to remove the TC template molecules. The products were alternately washed with H_2_O and ethanol multiple times. Ultimately, CdTe QDs@SiO_2_/N-CDs@MIPs were successfully obtained. The non-imprinted polymers (CdTe QDs@SiO_2_/N-CDs@NIPs) were prepared in the same way as above, but without the addition of template TC.

### 3.3. Fluorescent Detection of TC

Throughout the experimental procedure, all fluorescence measurements were conducted under uniform conditions, with the fluorescence spectra captured by a spectrofluorometer set to scan wavelengths from 350 nm to 750 nm, using an excitation wavelength of 345 nm. The photomultiplier tube voltage was adjusted to 600 V, the scanning rate was set at 1000 nm/min, and the slit widths for both emission and excitation were maintained at 10 nm. Both CdTe QDs@SiO_2_/N-CDs@MIPs and CdTe QDs@SiO_2_/N-CDs@NIPs were dissolved in distilled water to prepare a new stock solution with a concentration of 300 mg/L. The TC stock solution, with a concentration of 1.0 mmol/L, was refrigerated at 4.0 °C for subsequent use. At ambient temperature, the required amounts of CdTe QDs@SiO_2_/N-CDs@MIPs and CdTe QDs@SiO_2_/N-CDs@NIPs were introduced into separate 10 mL spectrophotometric tubes. Subsequently, the analyte standard solutions of specified concentrations were added in an orderly fashion. The solutions were allowed to stand in the spectrophotometric tubes for a designated period. A portion of each solution was then transferred to a quartz cuvette for the measurement and recording of fluorescence intensities.

### 3.4. Handling of Actual Samples

Milk and egg specimens were procured from a local grocery store. To eliminate the majority of proteins and fats present in these dairy and egg samples, 2.0 mL of milk and egg white were each placed into 10 mL centrifuge tubes. Subsequently, 2.0 mL of water and 1.0 mL of a 10% trichloroacetic acid solution were added to each tube. The mixtures were then subjected to ultrasonication for 30 min to ensure thorough mixing. Following this, the solutions were centrifuged at 10,000 rpm for 10 min to facilitate the separation of components within the samples. The supernatant from each tube was carefully transferred to a new centrifuge tube and the centrifugation process was repeated under identical conditions. Finally, the supernatant was passed through a 0.22 μm filter membrane to achieve clarification. The obtained samples were stored at 4.0 °C for the subsequent experiments. Finally, the DDW, referring to the method for detecting TC in the Section 3.3, was replaced with a solution of processed milk/eggs and then prepared as a test solution for testing. Each sample was tested for 6.0 times and the average value was taken.

### 3.5. High-Performance Liquid Chromatography (HPLC) Analysis Method

The High-Performance Liquid Chromatography (HPLC) protocol for the detection of tetracycline (TC) in eggs and milk was adapted from the 2006 master’s thesis of Tan Wang at Beijing University of Chemical Technology, with certain adjustments. Notably, a Shim-pack GIST C18 reversed-phase column (250 mm × 4.6 mm, 5 μm) was employed for the chromatographic separation at ambient temperature, utilizing an HPLC system (Agilent 1260 Infinity II, Agilent, Santa Clara, CA, USA) equipped with a UV detector set at 275 nm. The mobile phases were the acetic acid solution with pH = 4.0 and the acetonitrile solution, respectively. At 0–9.0 min, the proportion of acetonitrile increased from 15% to 30%, the proportion of acetonitrile remained unchanged at 30% at 9.0–13 min, then the proportion of acetonitrile increased to 85% at 13–15 min, and the proportion of acetonitrile returned to 15% at 15–16 min. The mobile phase flow rate was configured to 0.7 mL per minute, with an injection volume of the samples at 20 μL and the duration for detection set to 16 min. The techniques for preparing egg and milk samples mirrored those utilized in the fluorescence analysis. Ultimately, the actual samples underwent HPLC analysis for a total of six times, and the mean value was determined for reporting purposes.

## 4. Conclusions

In this work, a type of N-doped CD was used as the high-luminescence identification signal, and CdTe QDs@SiO_2_ was used as the stable fluorescent internal core. Consequently, a low-toxicity core–shell molecularly imprinted ratiometric fluorescence sensor, specifically CdTe QDs@SiO_2_/N-CDs@MIPs, was synthesized with success and subsequently deployed for the detection of TC in both milk and egg samples. This result demonstrated that the CdTe QDs@SiO_2_/N-CDs@MIPs showed high stability, excellent selectivity, and high sensitivity. Under optimal detection conditions, the CdTe QDs@SiO_2_/N-CDs@MIPs demonstrated a strong linear relationship with TC concentration, a fast response time, a low detection limit, and a clear visual effect on TC. In addition, the HPLC analysis results strongly proved the accuracy of the fluorescence analysis method proposed in this work. In summary, the CdTe QDs@SiO_2_/N-CDs@MIPs showed practical applications for TC detection in complex environments and achieved ideal results.

## Figures and Tables

**Table 1:** Fluorescence analysis and HPLC analysis and test results comparison of TC in milk and egg samples (*n* = 6).

	CdTe QDs@SiO_2_/N-CDs@MIPs				HPLC			
Sample	Add (μM)	Detected (μM)	Recovery Rate (%)	RSD (%)	Add (μM)	Detected (μM)	Recovery Rate (%)	RSD (%)
Milk	0	—	—	—	0	—	—	—
	10.00	9.96	99.60	1.86	10.00	10.18	101.80	1.24
	20.00	20.17	100.85	1.57	20.00	20.28	101.40	1.32
	40.00	40.18	100.45	1.16	40.00	40.15	100.38	1.09
Egg	0	—	—	—	0	—	—	—
	10.00	10.15	100.15	1.71	10.00	9.50	95.00	1.87
	20.00	19.98	99.90	1.38	20.00	19.68	98.40	1.56
	40.00	40.54	101.35	1.12	40.00	40.15	100.38	1.24

**Table 2:** Comparison of different methods for the detection of TC.

Method	Linear Range	Detection Time	Detection Limit	References
Aptamer-based method	0.011–0.056 mM	10 min	0.0225 mM	[44]
Fluorescence analysis	10–400 μM	10 min	6.0 μM	[45]
Electrochemical analysis	5.0–180 μM	—	2.0 μM	[46]
Spectrophotometric method	50–500 μM	40 min	2.4 μM	[47]
Voltammetric analysis method	0–95 μM	—	2.6 μM	[48]
Fluorescence analysis	0–33.23 μM	60 min	8.97–18.39 μM	[49]
Surface-enhanced Raman spectroscopy	9.0–56 μM	1.0 min	2.52 μM	[50]
Molecular imprinting method	2.0–120 μM	5.0 min	1.54 μM	[51]
CdTe QDs@SiO_2_/N-CDs@MIPs	0–140 μM	1.0 min	0.846 μM	This work

## Data Availability

Data are contained within the article.
